# Spatial Patterns of Movement of Dung Beetle Species in a Tropical Forest Suggest a New Trap Spacing for Dung Beetle Biodiversity Studies

**DOI:** 10.1371/journal.pone.0126112

**Published:** 2015-05-04

**Authors:** Pedro Giovâni da Silva, Malva Isabel Medina Hernández

**Affiliations:** Programa de Pós-Graduação em Ecologia, Departamento de Ecologia e Zoologia, Universidade Federal de Santa Catarina, Florianópolis, Santa Catarina, Brazil; Universidad Nacional Autonoma de Mexico, MEXICO

## Abstract

A primary goal of community ecologists is to understand the processes underlying the spatiotemporal patterns of species distribution. Understanding the dispersal process is of great interest in ecology because it is related to several mechanisms driving community structure. We investigated the mobility of dung beetles using mark-release-recapture technique, and tested the usefulness of the current recommendation for interaction distance between baited pitfall traps in the Brazilian Atlantic Forest. We found differences in mean movement rate between Scarabaeinae species, and between species with different sets of ecological traits. Large-diurnal-tunneler species showed greater mobility than did both large-nocturnal tunneler and roller species. Our results suggest that, based on the analyses of the whole community or the species with the highest number of recaptured individuals, the minimum distance of 50 m between pairs of baited pitfall traps proposed roughly 10 years ago is inadequate. Dung beetle species with different sets of ecological traits may differ in their dispersal ability, so we suggest a new minimum distance of 100 m between pairs of traps to minimize interference between baited pitfall traps for sampling copronecrophagous Scarabaeinae dung beetles.

## Introduction

Understanding the patterns of the spatiotemporal distribution of species is still a challenge for community ecologists. Dispersal is the capacity that organisms have to move over space, and is one of the four basic ecological processes driving such patterns [1]. This process is of great interest in ecology and evolution, because it is related to population and community dynamics, gene flow, speciation and extinction processes [2]. Dispersal is affected by several factors such as the ability to move through the landscape, perceptual resolution (shortest distance to detect resources), quality and distribution of the resource, and internal and external stimuli [3]. Species with dissimilar morphological and functional traits may have other resource requirements, and thus may have different rates of dispersal. Species with individuals who have higher dispersal ability may strongly alter the structure of local communities via patch dynamics or mass effects [4].

Dispersal was the key point for the development of metacommunity theory. A metacommunity is a set of local communities linked by the dispersal of multiple species [4, 5], and is primarily concerned with the role of dispersal between local communities in generating patterns of composition, abundance and species richness at multiple spatial scales. Understanding species dispersal processes is critical in current scenarios of habitat loss, fragmentation and global climate change [6]. The study of ability for movement by different organisms such as dung beetles (Coleoptera: Scarabaeidae: Scarabaeinae), which play key roles in the maintenance and restoration of ecosystems, is an important starting point for planning of conservation strategies.

Dung beetles are a very diverse group of detritus-feeding insects that have several ecological functions [7]. The diversity of the group is reflected in differences in body size [8, 9], body shape [10], resource relocation behavior for feeding and nesting [11, 12], and diel activity period [13, 14], for example. Dung beetle species may respond in different ways to alteration, disturbance, fragmentation, and loss of habitat [15], and as such they may be used as environmental indicators [16–18]. Several species from Neotropical forests exhibit varying degrees of habitat specificity, with many environmental specialists and generalists [19, 20]. Such diversity indicates that environmental changes and fragmentation may be barriers to dispersal of some dung beetle species [19, 21].

The community structure of dung beetles is strongly influenced by reproductive competition [22] over patchy and ephemeral food resources [23]. The high inter- and intraspecific competition coupled with random distribution and ephemerality of food suggest that dung beetles are probably good dispersers [24]. Studies on dispersal of Scarabaeinae dung beetles are few [25–30], however some authors suggest that there may be differences in dispersal ability among species, or among individuals within a species due to different interspecific and intraspecific species traits [26, 29, 31]. For example, male *Canthon cyanellus cyanellus* LeConte, 1859 were found to have a faster movement rate than females, and young-mature individuals moved more often than immature or old individuals in a Mexican dung beetle assemblage [29]. A diurnal large-bodied species, *Oxysternon conspicillatum* (Weber, 1801), was recaptured two days after release 1 km away in an Ecuadorian rain forest [26], a longer distance than that traveled by small-bodied species of *Onthophagus* Latreille, 1807 and *Canthon* Hoffmannsegg, 1817. Therefore, an understanding of the relative abilities of species to move within and between ecosystems may aid our understanding of how Scarabaeinae communities are structured both locally and regionally.

An important issue in the study of dung beetles is the lack of a standardized sampling protocol [32]. The sample design and the distance between traps indicted for sampling dung beetles vary widely, making it difficult to compare diversity patterns or community responses between studies. For instance, the movement of *Canthon acutus* Harold, 1868 was investigated in a mark-recapture experiment in which the authors observed that 95% of recaptured individuals were found within 26.2 m from traps [28]. These authors suggested that the minimum distance of 50 m between traps could reduce or eliminate interference between pairs of baited traps when sampling Scarabaeinae. However, this distance may vary between species due to foraging behavior or body size, for example. So, testing the proposed distance among baited traps based on the response of a single species may provide new information about the suitability of the suggested distance for other species and different ecosystems [33]. Establishing a standardized sampling protocol to limit or eliminate interference between pairs of baited traps is an important goal for dung beetle biodiversity studies [28, 32], because independence among samples is a basic premise in statistics analyses. Avoiding effects of pseudoreplication is a central issue in ecological studies [34], and the spatial distance between samples has several consequences on results [35] due to the intrinsic spatial variation that occurs in natural communities [36].

The aim of this study was to investigate the mobility of dung beetles, and to evaluate whether the current protocol of 50 m distance between baited pitfall traps is adequate to eliminate interference (or dependence) between traps in Scarabaeinae community studies. Based on the literature, we hypothesize that movement ability differs between Scarabaeinae species, and between individuals of each species within the same community due to various interspecific and intraspecific ecological traits (e.g. gender, age categories, body size, food relocation behavior, and diel activity period).

## Materials and Methods

### Study area

The study was developed in the Desterro Environmental Conservation Unit (UCAD), an environmental protected area of Atlantic Forest, located in Florianópolis, Santa Catarina Island, Brazil. The UCAD is located in the northwest (27°30’48”, 27°32’34” S; 48°29’38”, 48°30’42” W) of Santa Catarina Island and has an area totaling 491 ha of dense ombrophilous forest [37] with secondary vegetation. The climate is Cfa according to the Köppen-Geiger classification. The average annual temperature is 21.1°C (23.4°C in summer; 16.7°C in winter), and average annual rainfall is app. 1500 mm [37]. The terrain is mostly mountainous, with elevation ranging between 0–300 m.a.s.l. The altitude of sampling points ranged between 83–244 m.a.s.l.

### Sampling design

Dung beetles were sampled using baited pitfall traps during the spring and summer of 2013–2014 (November to March), which is the period of greatest abundance of this group in southern Brazil [38, 39]. Beetles were captured using plastic containers (15 cm diameter; 8 cm deep) with the cover cut to ¼ of its area to allow entry and avoid the escape of trapped insects (type A [40]). Rain guards were placed above the traps. Each trap was baited alternately between each 48 h sampling period with *app.* 20 g of human feces or rotten meat to attract coprophagous and necrophagous beetles, respectively [22, 40]. The baits were wrapped in a thin cloth and tied inside the trap for easy discarding, and to prevent manipulation by insects. Adjacent traps were fitted with the same bait in each sampling. The human samples used for the bait were from one of the authors (PGS).

**Fig 1:**
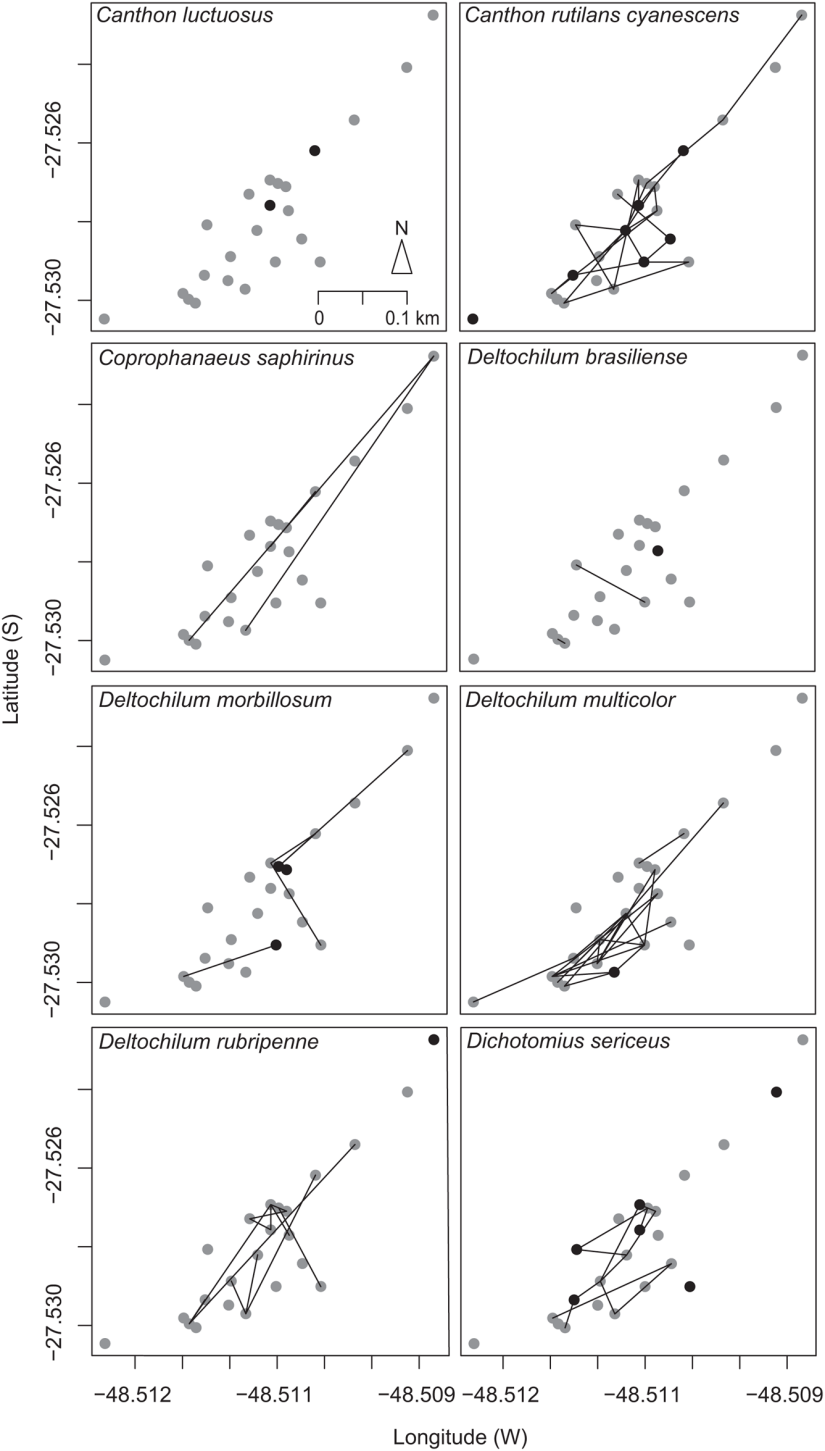
Movement patterns of dung beetle species. Circles depict the trap design. Black circles depict recaptures of individuals in the same trap. Each line segment depicts a dung beetle movement between two traps. Time between recaptures ranged from 5–87 d.

Dung beetles were sampled using three traps arranged along each of six linear and parallel transects; transects were spaced 50 m apart. Traps were spaced 10 m apart in the first and in the last transect, 25 m apart in the second and fifth transect, and 50 m apart in third and fourth transect. One additional trap was set 100 m before the first transect in an area with predominantly grassland and undergrowth vegetation, and with little presence of trees, and four additional traps were placed transversely to the latter transect at a distance of 100, 200, 350 and 500 m, for a total sampling effort of 23 traps (Fig 1, see Results). The distance between the first and the last trap was *app.* 1 km, and the spatial distribution of the traps was adjusted for land condition and trail access of the particular study area. We used different distances between traps in each transect (i.e. 10, 25 and 50 m) expecting that dung beetles recapture would be increased in closely spaced traps [28]. We calculated the area of study as the spatial distribution of traps using area formulas of geometric figures. We added 100 m to the sides of traps located in the extremes. The total study area was 0.23 km^2^.

All traps were baited at the first day of each sampling period, and insects were collected after 48 h. After beetles were removed, the baits were removed and properly discarded, and traps were dismounted. Captured dung beetles without marks were subsequently marked (see Mark-release-recapture section). The interval between each 48 h sampling period was 7.8 d on average (range 2–18 d due to climatic conditions) to allow the movement of individuals within the forest without bait interference. Nineteen samplings with duration of 48 h were carried out during the study period (November 2013 to March 2014). Marked beetles were resampled at each new 48 h sampling period, and unmarked beetles were marked and released the following day.

### Mark-release-recapture

After each 48 h sampling period, collected dung beetles were cleaned, identified, sexed, marked, and classified into one of three age classes. The identification was performed by an expert (Dr. Fernando Vaz-de-Mello, Universidade Federal de Mato Grosso, Brazil) from the Entomological Collection of the Federal University of Santa Catarina via comparison with previously identified species. Beetles were sexed and characters of sexual dimorphism were identified following species descriptions.

Individuals of each species were marked with a unique combination of points on the elytra and pronotum that allowed us to identify each specimen (S1 Fig). Marking was performed using an entomological needle with rough tip by scraping a thin layer of elytra and pronotum according to the distribution of the points. This technique is noninvasive, and the risk of being lost by the insect is lower that for some paints (previous laboratory observations). Marked individuals were kept in ventilated and moistened containers with leaf-litter, to be released the next day in the same place of capture (near the trap, the day after 48 h sampling period).

Age categories used were: (1) recently emerged or immature, (2) young-mature, and (3) old individuals. The assignment of age categories used the following criteria: aspect and hardness of the cuticle of the body, wearing stage of the teeth and spur of anterior legs, and clypeal teeth [29]. The relationship between the aspects of these characters with sexual maturity was previously established [29].

Dung beetle species were classified according to their behavioral guilds in dwellers (feed on and nest in the resource), rollers (build and roll food-balls over the soil until bury them), and tunnelers (bury portions of food under or next the resource) [41, 42]. The beetles were grouped into size categories as either small (≤ 1.5 cm length) or large (> 1.5 cm length). Sampled individuals of the genus *Canthonella* Chapin, 1930 and *Uroxys* Westwood, 1842 were not incorporated into this study, as marking them using the above technique was not possible due to the small size of individuals (< 0.5 cm length). The species were grouped into categories of diel activity periods as diurnal, nocturnal, or diurnal-nocturnal [13, 14].

The Instituto Chico Mendes de Conservação da Biodiversidade (ICMBio/MMA) issued the permits to collect specimens (permit #32333–3 to MIMH). The field study did not involve any endangered or protected species.

### Data analysis

#### Spatial patterns of movement

We used only recapture data to verify the movement patterns of dung beetles. The data set used in this study is available as S1 Dataset. We calculated the mean, median and maximum movement distance for each species. The movement of all individuals recaptured by species was shown schematically according to the spatial distribution of pitfall traps in the study area. Linear models, followed by residual analysis, were used to test for differences in movement rate between species and between individuals within each species in relation to gender, age, body size, relocation behavior, and diel activity. We calculated the movement rate (m/day) for each individual based on the observed data (distance moved during 24 h between samplings), multiplying the distance values by one (24 h) and dividing by the number of days between capture and recapture. Species that had no or few values for each category were excluded from the analyses. After the analysis, we conducted an *a posteriori* test to identify differences. The relation between movement distance (m) and time (d) was investigated using linear models for the entire community and individually for each species, with and without the use of recapture data at the same trap. Analyses were conducted using R 3.1.1 software [43] and associated packages.

#### Suitability of trap spacing

Nonlinear regression analyses were performed to verify the movement distance during 48 and 96 h using SigmaPlot 10.0 program. We estimated the linear distance traveled by dung beetles (in a straight line between two traps) in 48 and 96 h, with the aim of establishing a minimum distance between baited pitfall traps that maximizes sampling efficiency, reducing the sampling area and the possible interaction between traps [32]. Such periods are commonly used in studies of these fauna. We estimated the distance traveled by each individual during 48 and 96 h based on the observed data (distance moved during the period between each 48 h sampling period), multiplying the distance values by two (48 h) or four (96 h) and dividing by the number of days between capture and recapture of each individual. After that, we calculated the number of individuals recaptured by each distance category (0–10, 11–25, 26–50, 51–75, 76–100, 101–150, 151–300, 301–500, 501–750, and 751–900 m) and then divided the number of recaptures by the number of individuals recaptured in the smallest distance class. This proportion was used to reduce the effect of differences in beetle behavior [28], because although there was a long period between 48 h sampling periods, there were a large number of recaptures in the same trap, indicating that many individuals remained foraging or were buried near the traps for long periods. Our results showed that the 0–10 m category was represented by recaptures only at the same trap. In the nonlinear regression analysis, we used data on the proportion of individuals recaptured in each distance category and the minimum value of each category to avoid overestimation of the distance traveled by beetles. We calculated the definite integral of nonlinear regression analysis and determined the distance corresponding to 95 and 99% of the area under the curve [28]. This distance is the estimated radius of movement distance over a certain period of time in which 95 and 99% of individuals would be captured. These analyses were conducted for the entire community, and also for the species with the highest number of recaptures (to test the effect of distance on the proportion of recaptured individuals). We analyzed rotten meat and feces separately, because these baits likely attract dung beetle species differently [44]. The radius of bait attraction and the distance moved by beetles without baits should be taken into account in establishing a distance between traps that minimizes interference between pairs of baited pitfall traps.

The geographical coordinates of each trap were recorded using a hand-held GPS at ground level. The distance between each pair of traps was corrected for differences in elevation using the triangle-rectangle formula (Pythagorean Theorem) to more accurately estimate the straight-line distance traveled by dung beetles.

## Results

A total of 1,806 individuals belonging to 17 species were marked and released (S1 Table). *Canthon rutilans cyanescens* Harold, 1868, *Dichotomius sericeus* (Harold, 1867), and *Deltochilum morbillosum* Burmeister, 1848 were the species with the highest number of marked and released individuals. A total of 112 (6.2%) individuals (58 males and 54 females) belonging to eight species were recaptured (Table 1) with an overall recapture rate of 6.3% (range 1.5–22%). Twelve individuals (seven males and five females) were recaptured twice and two other individuals (one male and one female) three times. Three species were classified as small and four as large. We recaptured six rollers and two tunnelers. Young-mature individuals accounted for almost 60% of recaptured individuals. Only three individuals of *Deltochilum rubripenne* (Gory, 1831) were classified as old individuals. We recaptured three diurnal, three diurnal-nocturnal, and two nocturnal species. Only *Canthon luctuosus* Harold, 1868 showed no movement between traps (Fig 1). For individuals recaptured at the same trap, the average time to recapture was 14.2 d (range 5–67 d).

**Table 1:** Number of marked and recaptured individuals by gender and age categories, with movement values and time between recaptures for Scarabaeinae species.

Species^a^	Individuals			Gender		Age			Movement (m)					Time (range) in days
	Mk	Rc	%	M	F	IM	MA	OL	MMR	Same	Me	Md	Max	
A. *Canthon luctuosus* Harold, 1868 ^S,R,DN^	133	2	1.5	0	2	1	1	0	0	2	-	-	-	7(7–7)
B. *Canthon rutilans cyanescens* Harold, 1868 ^S,R,D^	677	35	5.2	17	18	23	12	0	9.82	11	143.51	85.95	504.72	24.7(7–82)
C. *Coprophanaeus saphirinus* (Sturm, 1826) ^L,T,D^	61	3	4.9	2	1	1	2	0	36.03	0	607.79	807.98	852.74	16.6(14–22)
D. *Deltochilum brasiliense* (Castelnau, 1840) ^L,R,N^	18	3	16.7	0	3	1	2	0	2.44	1	70.59	70.59	127.84	19(7–43)
E. *Deltochilum morbillosum* Burmeister, 1848 ^S,R,DN^	168	9	5.4	7	2	4	5	0	3.96	3	194.74	186.93	358.43	40.7(6–87)
F. *Deltochilum multicolor* Balthasar, 1939 ^L,R,DN^	100	22	22.0	10	12	6	16	0	12.61	1	205.98	206.68	551.76	24.8(7–74)
G. *Deltochilum rubripenne* (Gory, 1831) ^L,R,D^	131	16	12.2	10	6	1	12	3	5.17	1	260.22	226.39	614.79	57.6(14–94)
H. *Dichotomius sericeus* (Harold, 1867) ^L,T,N^	451	22	4.9	8	14	5	17	0	7.21	10	109.93	88.40	222.88	18.3(5–81)
Total	1606	112	6.2	54	58	42	67	3						

Marked (Mk) and recaptured (Rc) dung beetle individuals. %: recapture rate. Gender: male (M) and female (M). Age categories: immature (IM), young-mature (MA), and old (OL) individuals. Movement (m): mean movement rate (MMR [m/d]), number of individuals recaptured at the same trap (Same), mean (Me), median (Md) and maximum (Max) movement distance for individuals that did move between traps.

^a^Size categories: small (S, ≤ 1.5 cm) and large (L, > 1.5 cm). Behavioral categories: roller (R) and tunneler (T) species. Diel activity: diurnal (D), nocturnal (N), and diurnal-nocturnal (DN) species.

### Spatial patterns of movement

The spatial patterns of movement of dung beetles may be seen in Fig 1. *Canthon r*. *cyanescens* moved across the entire study area (Fig 1). Recaptured *C*. *r*. *cyanescens* were represented by similar numbers of males and females, mostly immature individuals. *Coprophanaeus saphirinus* (Sturm, 1826) showed the furthest movements among dung beetle species (*app.* 850 m in straight line) (Fig 1), and we found similar numbers of recaptured males and females, and immature and young-mature individuals. Female *Deltochilum brasiliense* (Castelnau, 1840), a large-bodied roller species, moved shorter distances (Fig 1). Similar numbers of immature and young-mature male *D*. *morbillosum* were found (Fig 1). *Deltochilum multicolor* Balthasar, 1939 showed a concentration of movement at the southeast portion of the sampling area (Fig 1). It was the most recaptured species (22% of total recapture), and was represented by similar number of males and females, mostly young-mature individuals. *Deltochilum multicolor* was the only species that moved through the area with predominantly grassland and undergrowth vegetation and with little presence of trees (between the first trap and the first transect, *app.* 100 m away). *Deltochilum rubripenne* concentrated movement in the middle portion of the sampling area (Fig 1), and was represented mainly by young-mature males. *Deltochilum rubripenne* showed the second longest maximum movement distance (*app.* 614 m in straight line). *Dichotomius sericeus* concentrated movement between the six transects located in the middle of the sampling area (Fig 1). *Dichotomius sericeus* was most represented by young-mature females.

The mean movement rate varied among dung beetle species (*F* = 3.85, *P* = 0.002). *Coprophanaeus saphirinus* showed a higher movement rate than other species (S2 Fig). There was no difference in movement rate between species with different periods of diel activity (*F* = 0.55, *P* = 0.57), body size categories (*F* = 0.30, *P* = 0.58), or relocation behaviors (*F* = 1.31, *P* = 0.25). There was a significant interaction between relocation behavior and diel activity period (*F* = 4.57, *P* = 0.002). Diurnal-tunneler species showed the highest mean movement rate and differed from nocturnal-tunneler, diurnal-roller and nocturnal-roller species (S3 Fig). The interaction between body size, diel activity and relocation behavior period was also significant (*F* = 3.85, *P* = 0.002) and showed that large-diurnal tunneler species had greater movement rate than did other species (S4 Fig). There were no differences in mean movement rate for the remaining factors and interactions (S2 Table).

**Fig 2:**
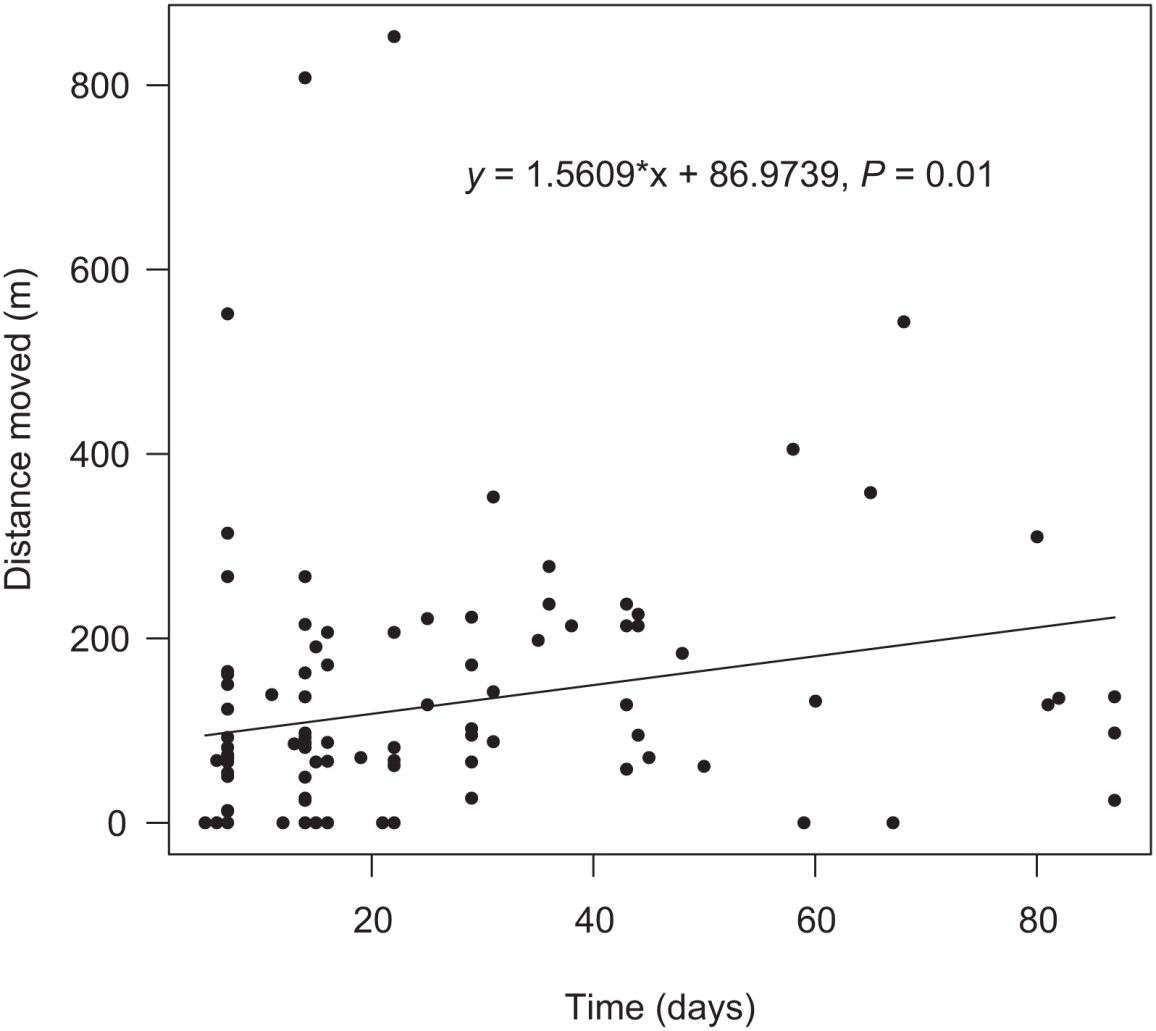
Linear model of movement distance (m) and time (d) for recaptured Scarabaeinae, including recaptures at same trap.

We observed a positive and significant relationship between time and distance moved by dung beetles, including recaptures at the same trap (*F* = 5.70, *P* = 0.01; Fig 2). This pattern was not observed when data from recaptures at the same trap were excluded (S5 Fig). Using the equation of the linear model (Fig 2), we found that the estimated movement distance traveled in 48 h by dung beetles was 90 m. For 96 h, the distance was 93 m. Movement distance was positively related to time only for *C*. *r*. *cyanescens* (*F* = 5.82, *P* = 0.02) (including recaptures at the same trap), in which the estimated distance traveled in 48 h was 79 m.

### Suitability of trap spacing

**Fig 3:**
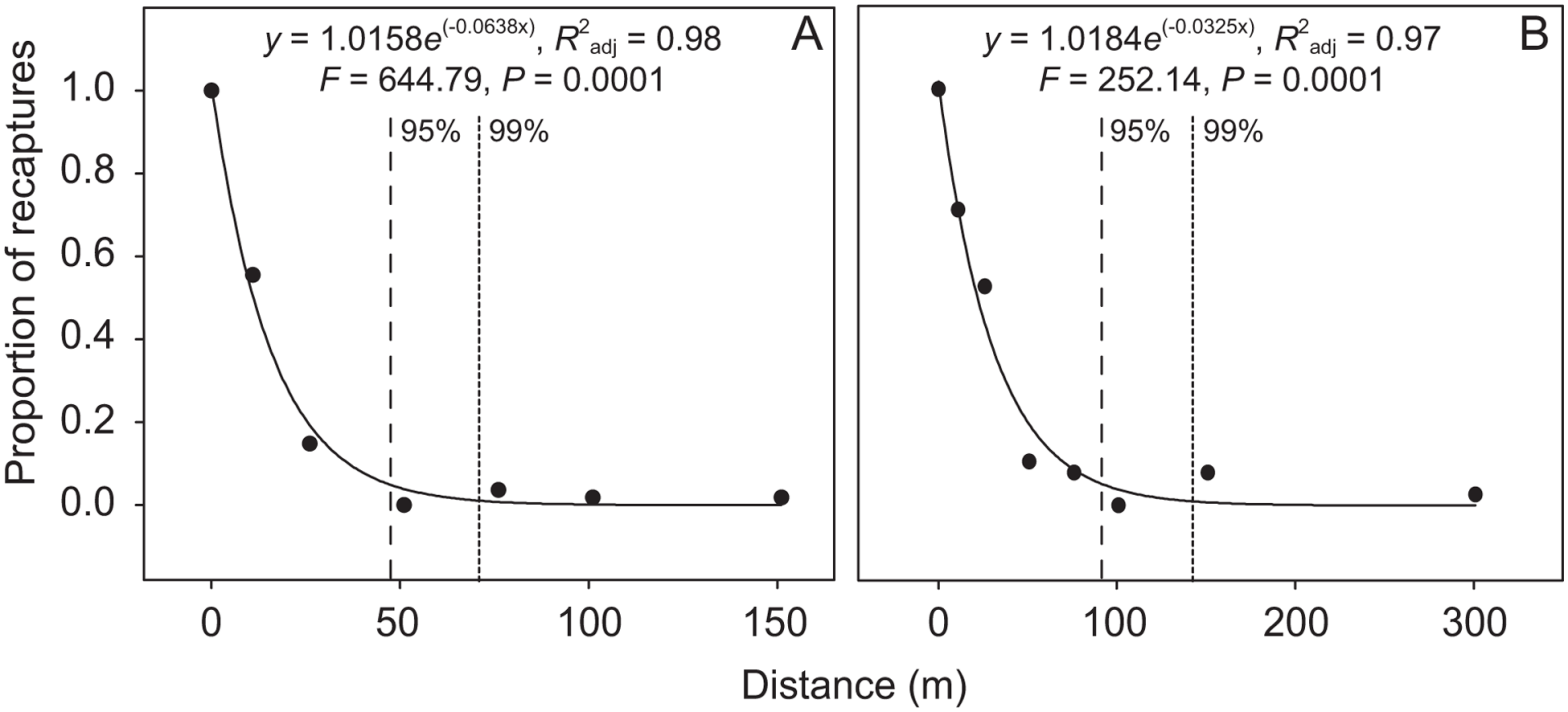
Proportion of recaptured Scarabaeinae with increasing distance for estimated time periods of 48 (A) and 96 h (B). Each proportion was normalized by the number of beetles recaptured in the smallest distance category (0–10 m). Dashed and dotted lines represent the radius for 95 and 99% of recaptured individuals, respectively. The distance is an estimate based on the distance traveled by beetles during longer periods.

**Fig 4:**
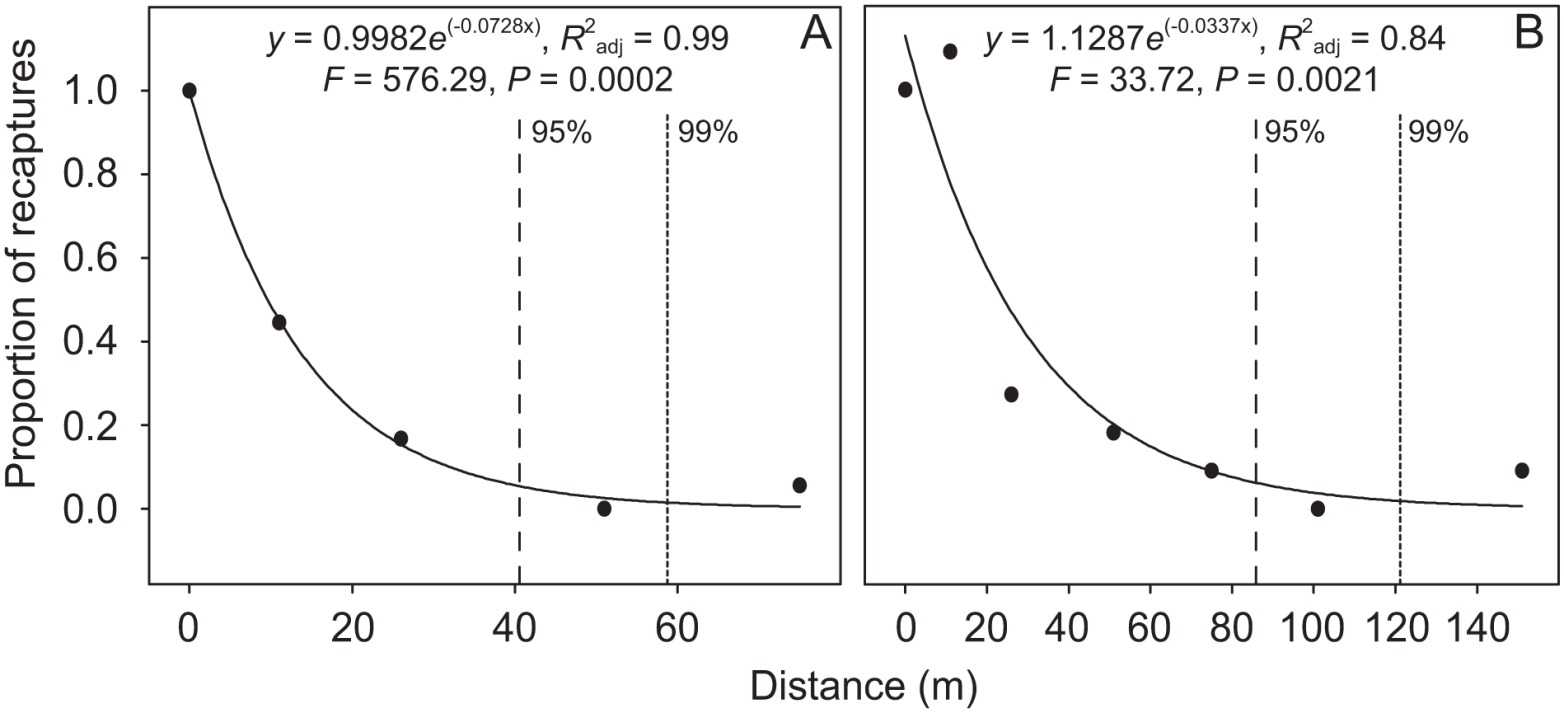
Proportion of recaptured *Canthon rutilans cyanescens* with increasing distance for estimated time periods of 48 (A) and 96 h (B). Each proportion was normalized by the number of beetles recaptured in the smallest distance category (0–10 m). Dashed and dotted lines represent the radius for 95 and 99% of recaptured individuals, respectively. The distance is an estimate based on the distance traveled by beetles during longer periods.

The nonlinear regression analysis showed that the estimated proportion of recaptured beetles decreased rapidly with increasing distance in both 48 h (*y* = 1.0158*e* ^(-0.0638x)^, *R* ^2^_adj_ = 0.98, *F* = 644.79, *P* = 0.0001; Fig 3A) and 96 h (*y* = 1.0184*e* ^(-0.0325x)^, *R* ^2^_adj_ = 0.97, *F* = 252.14, *P* = 0.0001; Fig 3B). By calculating the area under the curve, we estimated a movement radius of 47 m where 95% of the beetles would be captured within 48 h (Fig 3A). For this time period, 99% of individuals would be recaptured within a radius of 72 m. The movement radius in which 95% of the beetles would be recaptured in 96 h was estimated at 92 m (Fig 3B). Ninety-nine percent of individuals would be recaptured within 143 m for this time period. Using only the movement data of *C*. *r*. *cyanescens*, the distances where 95 and 99% of individuals would be recaptured at 48 h were 40.5 and 59 m (*y* = 0.9982*e* ^(-0.0720x)^, *R* ^2^_adj_ = 0.99, *F* = 576.29, *P* = 0.0002; Fig 4A), respectively. At 96 h, the distances where 95 and 99% of individuals would be recaptured were 85.3 and 122 m (*y* = 1.1287*e* ^(-0.0337x)^, *R* ^2^_adj_ = 0.84, *F* = 33.72, *P* = 0.0021; Fig 4B), respectively.

Similar results were found when we analyzed the two types of baits separately. For rotten meat, nonlinear regression analysis showed that the proportion of recaptured dung beetles decreased with increasing distance in both 48 h (*y* = 1.0203*e* ^(-0.0672x)^, *R* ^2^_adj_ = 0.97, *F* = 291.27, *P* = 0.0001; S6A Fig) and 96 h (*y* = 0.9878*e* ^(-0.0312x)^, *R* ^2^_adj_ = 0.92, *F* = 93.66, *P* = 0.0001; S6B Fig). By calculating the area under the curve, we estimated a movement radius of 45.5 m and 68 m in which 95% and 99% of the beetles would be captured within 48 h, respectively (S6A Fig). For the 96 h sampling period, we estimated a movement radius of 96 m and 146 m in which 95% and 99% of the beetles would be captured, respectively (S6B Fig).

For human feces, nonlinear regression analysis showed no significance for the 48 h sampling period (*F* = 60.66, *P* = 0.081). For the 96 h sampling period, the proportion of recaptured dung beetles also decreased with increasing distance (*y* = 1.0356*e* ^(-0.0435x)^, *R* ^2^_adj_ = 0.84, *F* = 23.05, *P* = 0.017), and we estimated a movement radius of 57 m and 70.5 m in which 95% and 99% of the beetles would be captured, respectively.

## Discussion

### Spatial patterns of movement

This study assessed the mobility of a dung beetle assemblage. Our results indicated differences in the movement rate of species with different sets of ecological traits, including food resource relocation behavior, body size, and diel activity period. This finding may have implications for how Scarabaeinae assemblages are structured locally and regionally. Due to low recapture rate of some species, our results should be taken with caution, however we hope the data will be valuable for generation of new hypotheses for contribution to an area with large knowledge gap, including dung beetle dispersal ability and associated implications for structuring communities.

The low recapture rate among Scarabaeinae [29] and related groups (e.g. Aphodiinae [45]) is common in dispersal studies (see S3 Table and references therein). Our results, however, show that recapture rates vary between dung beetle species (1.5–22%), and the most abundant species does not always have the highest recapture rate (e.g. [45]). These results imply that some species with high recapture rates may simply have more limited spatial distribution than others, which may be related to variation in environmental characteristics at small spatial scales, availability of particular food resources, or limited dispersal. On the other hand, low recapture rates may be related to high dispersal rates, whereby species fly longer distances due to random distribution and ephemerality of food resources. Some dung beetle species also remain buried for long time periods while they are rearing offspring [46], which may be associated with the high average time between recaptures (23.6 d, range 5–87 d). Perhaps the release of a large quantity of individuals of different species at the same time may provide better Scarabaeinae recapture rates.

Some dung beetle traits, such as body size (or biomass) and relocation behavior, have been identified as important for investigating the response of Scarabaeinae to tropical forest conversion [47, 48], forest fragmentation [19, 49, 50], and ecological function performance [51–53]. We expected the same for differences in dispersal ability among dung beetle species, where some traits would contribute more or less to high versus low movement rates of species. Identifying these traits is crucial to our understanding of the role of dispersal in structuring communities both locally and regionally.

Body size and wing loading are correlated [49], and large-bodied dung beetles with high wing loading usually use cruise flight foraging strategy [26, 31, 49], which allows them to disperse further [49]. In contrast, small-bodied dung beetles with low wing loading usually use a perching strategy [26, 31, 54], which may restrict the ability to travel large distances. The tribe Phanaeini (represented by the genus *Coprophanaeus* Olsoufieff, 1924 in our study) has the largest dung beetles of the Neotropical region, and generally its species are cruising beetles [55]. *Coprophanaeus saphirinus* showed the highest movement rate and maximum distance traveled in our study (*app.* 850 m in straight line). The interaction of body size, diel activity and relocation behavior was important, and large-diurnal-tunnelers showed greater mean movement rate than other species. Thus, different sets of ecological traits may contribute to differences in the dispersal ability of dung beetles. These findings have several implications in the context of metacommunity theory, mainly for models in which dispersal has a key role, such as mass effects (high dispersal), species sorting (intermediate dispersal) and dispersal limitation (low dispersal) [4, 56]. Ecologists often mix “oranges with apples” [57], i.e. we expect that all species respond the same way to environmental and spatial processes, which may be not true. Some species traits, such as body size, activity period, and relocation behavior may play an important role in distinguishing dung beetle species that are more influenced by environmental or spatial processes (see [58]). In other words, some traits may facilitate dispersal of dung beetle species, so that they respond differently to different ecological processes.

Different flight periods in dung beetles may have evolved in correlation with defecation patterns of mammals, and the body size of dung beetles has great importance in the daily activity period of the species [59]. There are diurnal, nocturnal, diurnal-nocturnal, and crepuscular species, and some have restricted flight time while others fly for long time periods [55]. Large tunnelers are generally nocturnal and small rollers are generally diurnal [42], but there are exceptions, such as *C*. *saphirinus*, that are a diurnal and large-bodied species.

In general, a temperature range between 25–42°C is optimal for dung beetle flight [60]. The average maximum and minimum temperatures in the study region vary between 10–16 and 35–38°C, respectively, between November and March [61]. Thus, nocturnal species may have a higher limitation on flight due to temperature conditions being more unfavorable at night. Energy expenditure may also be higher for nocturnal species than for diurnal species, resulting in shorter flights. This hypothesis is among the demands for a better understanding of the relationship between body temperature and activity period of dung beetles [60], since several species may increase body temperature during cold periods in order to fly [62]. However, we have no thermoregulation data on species sampled in our study.

The sun, the moon, celestial polarization, and the milky-way serve as guides for dung beetles [63], and both diurnal and nocturnal species have eyes adapted for vision in dim light [64]. Photoreceptor mechanisms of nocturnal beetles show different responses depending on the speed of flight [64]. Cruising beetles can be divided into fast (typical for diurnal species) and slow fliers (typical for nocturnal species) [55]. The tribe Phanaeini has fast flier species that cover a great extent of terrain during flight, which may last for many hours or shorter periods per day, as in some *Coprophanaeus* species [55]. Flights with lower speeds provide greater visual resolution of obstacles, but leave nocturnal dung beetles at higher risk of predation. Flights at higher speeds on the other hand cause a decline in the control of flight performance [65] making nocturnal beetles clumsy fliers [64]. Most nocturnal species fly close to the dung pats but not onto them; individuals land at a distance from the resource and walk to it, suggesting that “quick and dirty” is the best strategy for nocturnal dung beetle foraging flights [66]. In Neotropical forests, nocturnal dung beetles have flight speeds much lower than diurnal species to reduce energy costs and maximize the amount of time spent searching for food [55]. Due to the fact that the canopy does not easily allow the viewing of celestial cues and light rays, flights inside the forest may be a major difficulty for nocturnal species to move both on the ground and during flight. This fact may contribute to nocturnal species to move smaller distances than diurnal species within the forest, which can be more easily guided by light rays during the day.

Another important issue associated with the high movement rates found for large-diurnal tunneler species is the predation pressure that one may expect to be more important at certain times of day, depending on the diel activity of predatory species. For nocturnal species, short periods of flight may be expected to reduce the pressure of visual predators. The opposite is expected for diurnal colorful species, such as *C*. *saphirinus*, which travels greater distances by avoidance of visual predators due to body coloration that camouflages or advertises their toxicity to predators. Among the predators we can generally cite some birds (e.g. owls), mammals (e.g. bats), spiders and species of beetles from the families Carabidae and Staphylinidae [55, 67, 68]. However, predation on dung beetles still needs to be further investigated, because its effect on species behavior or community structure may be minimal or insignificant, as suggested by the meager available information [69].

Ecologically, a species is a set of individuals sharing similar traits that determine where and when they can live and how they interact with other species [70, 71]. Individually, a trait may not be enough to differentiate the species response to environmental and spatial processes, because two species that respond differently to these processes can share this unique trait. For example, not all large-sized Scarabaeinae species are expected to have a great extent of movement, because they can have differences in thermoregulation ability, flight speed or typical activity time. Therefore, investigating the interaction of some key species traits may be useful for understanding how and why species have a spatially structured distribution.

### Suitability of trap spacing

The proportion of individuals recaptured with increasing estimated distance showed that the 50 m between baited traps for sampling dung beetles previously proposed [28] is inadequate for species from an assemblage in the Brazilian Atlantic Forest. Our results showed that the longer the time between recaptures, the higher the distance traveled by dung beetles, a result supported by other authors [29]. When we analyzed recapture of individuals of all species, the radius of the effective sampling area (ESA) was estimated at 47 m and 92 m for 48 h and 96 h, in which 95% of individuals would be captured. When we analyzed only the recapture of *C*. *r*. *cyanescens*, the radius of the ESA where 95% of individuals would be captured in 48 h and 96 h was 40.5 m and 85.3 m, respectively. Our results were also supported when we analyzed the two bait types separately.

Based on recapture rate of *C*. *acutus*, some authors [28] recommended a sample design of linear transects with 10 baited pitfall traps spaced at least 50 m apart for sampling dung beetles, a method adopted by several authors. We agree with these authors [28] about the sample design, but according to our results, we suggest a new minimum distance of 100 m between pairs of baited traps for sampling Scarabaeinae during 48 h, taking into account bait attraction and the estimated distance traveled by beetles during this period. When possible, we recommend the use of greater distances between baited pitfall traps, as has been done in some studies in the Brazilian Amazonia (e.g. 200 m between pitfall traps [21, 72]). With this larger spacing between baited traps, we are not trying to use the traps as “true replicates” (see [34, 73]). Pseudoreplication is a problem among biodiversity studies in tropical forests, and obtaining real spatial variation within replicates is necessary for ecological studies [34]; this can be attained with adequate sampling design.

According to the vast literature, human feces remains the most effective bait for the attraction of dung beetles in the Neotropical region [74], even compared with feces of native mammals [75, 76] or other baits like rotten meat or decaying fruit [44]. Human-pig mixes may be a promising alternative for sampling Scarabaeinae. However, obtaining human feces is much easier than pig feces, and human and human-pig feces show similar attractiveness [77]. The use of human feces and decaying meat as bait is useful for attracting dung beetle species with different food preferences (copronecrophagous species) in the Neotropical region. The removal of insects and renewal of baits may be performed daily if necessary (e.g. [20, 21]), and series of sampling of 48 h may increase the sampling sufficiency.

Increasing sampling time must be followed by an increase in spacing between traps. We understand that the sample design may be restricted by physical characteristics of the study site [28] or may be spatially distributed according to the purpose of the study. Our new proposed trap spacing is suitable for sites with at least 1000 m in length, including border areas. If a site has this minimal size, then the new spacing can also be adopted for open areas. The use of linear design may be suitable for smaller sites, by placing two transects of five traps each or reducing the number of traps and conducting sampling series so there is at least a sampling effort of 10 traps, which seems an appropriate number of traps for the construction of sample sufficiency curves (see [78, 79]). For studies investigating the effect of fragmentation (e.g. fragment size), the use of smaller distances between baited pitfall traps should be adopted (e.g. [18]) according to the design of sample area or purpose of the study. Our new trap spacing may be suitable for investigating the response of dung beetles to ecological processes that require a considerable spatial extent to reveal their effects (e.g. [80]), such as environmental filtering and spatial processes (i.e. high dispersal or dispersal limitation).

Dung beetles perform several ecological functions important for the maintenance of ecosystems [7]. These insects may be used for understanding and monitoring the relationship between human-driven disturbance, patterns of biodiversity and ecosystem functioning [15, 21, 53] when they are properly sampled [28]. Knowing the movement process of dung beetles is critical to understand how communities are structured both locally and in the metacommunity [80]. Species with different sets of ecological traits may have different movement patterns and thus, they may influence local communities differently.

The use of standardized sampling protocols is essential to generate information necessary to investigate the processes that sustain biodiversity and ecosystem functioning [28], and to make the results comparable between studies conducted in different regions of the world [32]. Based on our estimates, we suggest a new minimum distance of 100 m between traps to minimize the dependence between pairs of baited pitfall traps for sampling copronecrophagous Scarabaeinae dung beetles in Neotropical forests. The use of this new minimum distance is also encouraged for other types of environments. The results of our and other studies (S3 Table) suggest that several species of dung beetles have high dispersal ability, which is related to some species traits and may be little known due to the difficulty of conducting such studies due to spatial limitations of the sampling design (or area) and the low recapture rate of this fauna.

## Supporting Information

S1 Dataset Dataset used to test for differences in movement rate by Scarabaeinae dung beetle species. Samplings were performed in Brazilian Atlantic Forest, Santa Catarina, Brazil using baited pitfall traps from November 2013 to March 2014.
(XLSX)

S1 Fig Marking points used in mark-release-recapture experiment. Distribution of marking points on elytra and pronotum used to mark Scarabaeinae dung beetles (A) and example of number #108 on an individual of *Dichotomius sericeus* (B).
(EPS)

S2 Fig Boxplots of movement rate of dung beetle species. Letters in x-axis indicate species names in Table 1. Gray asterisks represent the mean movement rate.
(EPS)

S3 Fig Boxplots of movement rate of dung beetle species with different reproductive behavior and diel activity periods. Gray asterisks represent the mean movement rate.
(EPS)

S4 Fig Boxplots of movement rate of dung beetle species with different body size, diel activity period and relocation behavior. Gray asterisks represent the mean movement rate. D: diurnal; DN: diurnal-nocturnal; N: nocturnal.
(EPS)

S5 Fig Linear model between movement distance and time for recaptured individuals of Scarabaeinae, excluding recaptures at same trap. Distance in meters and time in days.
(EPS)

S6 Fig Proportion of recaptured individuals of Scarabaeinae with increasing distance for estimated time periods of 48 (A) and 96 h (B) using rotten meat bait. Each proportion was normalized by the number of beetles recaptured in the smallest distance category (0–10 m). Dashed and dotted lines represent the radius for 95 and 99% of recaptured individuals, respectively.
(EPS)

S1 Table Summary of mark-release-recapture experiment. Number of marked and recaptured individuals, number of males and females, number of immature, young-mature and old individuals of dung beetles sampled in the Atlantic Forest in southern Brazil.
(XLSX)

S2 Table Results of linear models comparing movement rate between dung beetle species or individuals of each species. DF: degrees of freedom. Significant *P* values are in bold.
(XLSX)

S3 Table Studies of dung beetle dispersal using mark-release-recapture conducted in the Neotropical region. Na: not applicable or not informed. Mean: mean movement distance. Max: maximum movement distance. Time: days.
(XLSX)
